# Prevalence of Surgical Procedures at Symptomatic Onset of Prion Disease

**DOI:** 10.1001/jamanetworkopen.2022.1556

**Published:** 2022-03-09

**Authors:** Amanda L. Porter, Christian C. Prusinski, Evelyn Lazar, Clara Yuh, Robert C. Bucelli, Brian S. Appleby, Gregory S. Day

**Affiliations:** 1 Department of Neurology, Mayo Clinic Florida, Jacksonville; 2 Santa Clara Valley Medical Center, Santa Clara, California; 3 Department of Neurology, Washington University School of Medicine, St Louis, Missouri; 4 National Prion Disease Pathology Surveillance Center, Case Western Reserve, Cleveland, Ohio

## Abstract

This case-control study examines the frequency of invasive procedures at the onset of prion disease symptoms to determine the scope of the risk of contamination to future patients.

## Introduction

Creutzfeldt-Jakob disease (CJD) is a rapidly progressive fatal prion disease. Although most cases are sporadic or inherited, prions may be transmitted via contaminated tissues or durable medical equipment.^1,2^ The risk of iatrogenic transmission is highest following procedures involving the central nervous system, where prion burden is highest.^2,3^ However, experimental models suggest that transmission may occur following contact with other tissues (eg, nasal mucosa, lung, lymph nodes, and spleen).^4^ If these models are accurate, surgical procedures involving these tissues may pose a risk to future patients. To evaluate the potential scope of this problem, we determined the frequency of invasive procedures performed in patients with CJD at multiple tertiary care centers.

## Methods

Protocols for this case-control study were approved by the Mayo Clinic institutional review board. A waiver of consent was granted for the use of retrospective, deidentified data. This study follows the Strengthening the Reporting of Observational Studies in Epidemiology (STROBE) reporting guideline. An automated search of Mayo Clinic records identified 252 of 1 843 675 patients with diagnostic evaluation including “CJD” or “prion disease” evaluated from January 2014 (the time at which specific biomarkers of prion disease were incorporated within clinical practice) to February 2021 at Mayo Clinic locations in Rochester, Minnesota; Jacksonville, Florida; and Phoenix, Arizona. Fourteen patients at Washington University (Saint Louis, Missouri) were enrolled from February 2016 to December 2019 within prospective studies of rapid progressive dementia. Available records were dual-reviewed (April 2021) to identify patients who met criteria for probable CJD (neuropsychiatric disorder with positive cerebrospinal fluid real-time quaking-induced recovery assays; or rapidly progressive dementia with 1 or more of the following signs and symptoms: myoclonus, visual or cerebellar signs, pyramidal or extrapyramidal signs, akinetic mutism, and consistent brain magnetic resonance imaging; 71 patients) or definite CJD (pathologically or genetically confirmed, 50 patients)^5^ and to capture surgical procedures. Procedures performed within 1 year of the onset of symptoms attributed to CJD were counted to include the presymptomatic period associated with latent prion accumulation. Procedures were stratified by risk of prion contamination of instruments.^3,4^ Statistical analysis was performed using SPSS statistical software version 28.0 (IBM), using Pearson χ^2^ tests for categorical variables and Mann-Whitney U tests for continuous variables to evaluate the association between patient-specific factors and procedures. Significance was set at *P* < .10 due to the exploratory nature of this analysis. Data were analyzed from March 2021 to June 2021.

## Results

In total, 26 of 121 patients (21%) (63 female patients [52%]; median [range] age, 65.4 [21.9 to 81.5] years) with CJD underwent 55 procedures, including high-risk procedures in 2 patients with neuropathologically proven CJD (Table 1). Procedures were more frequent in patients with a history of arthritis (odds ratio [CI] 5.58 [1.16-26.7], *P* = .02) and possibly less frequent in patients with behavioral symptoms or signs at presentation (odds ratio [CI] 0.43 [0.16-1.17]. *P* = .093) (Table 2). Median times from symptom onset to brain magnetic resonance imaging and electroencephalogram were greater in patients who underwent procedures, suggesting that diagnostic delays were associated with procedures. Seventeen of 32 procedures (53%) were performed in the months prior to symptomatic onset (median [range,] −5.4 [−0.2 to −10.8] months). Appropriate procedural precautions were observed in 1 patient.

**Table 1.** Surgical Procedures Performed in Patients With Creutzfeldt-Jakob Disease Using Durable Instruments, Stratified by Risk of Contamination of Instruments With Prions^a^

Procedures	Indication	Procedures, No. (%) (N = 55)
High risk		2 (4)
Ophthalmic artery aneurysm clipping	Unruptured aneurysm	1 (50)
Brain biopsy	Diagnostic	1 (50)
Moderate risk		12 (22)
Joint replacement (knee or hip)	Fracture, osteoarthritis	4 (33)
Arthroscopy	Osteoarthritis	1 (8)
Bilateral carpal tunnel release	Carpal tunnel syndrome	1 (8)
Cholecystectomy (laparoscopic)	Cholelithiasis	1 (8)
Hernia repair and gastroplasty (laparoscopic)	Dysphagia	1 (8)
Laminectomy and facetectomy	Spinal stenosis	1 (8)
Laparoscopic salpingo-oophorectomy	Ovarian mass	1 (8)
Open-reduction internal fixation	Tibia or fibula fracture	1 (8)
Rotator cuff repair (laparoscopic)	Rotator cuff tear	1 (8)
Low risk		18 (33)
Endoscopy, gastrointestinal	Screening with or without polypectomy	9 (50)
Oral and maxillofacial surgery	Dental grafting	4 (22)
Nasal, laryngoscopy	Dysarthria, laryngitis	2 (11)
Bronchoscopy	Pneumonia	1 (6)
Cystoscopy	Retention	1 (6)
Ophthalmic surgery	Cataract removal	1 (6)
Negligible risk		23 (42)
Biopsy, skin or lip	Diagnostic or therapeutic	5 (22)
Joint injection or aspiration	Diagnostic or therapeutic	5 (22)
Angiography with or without stenting	Diagnostic or therapeutic	3 (13)
Central venous catheter placement	Therapeutic	2 (9)
Endotracheal intubation	Therapeutic	2 (9)
Acupuncture	Therapeutic	1 (4)
Implantable loop recorder placement	Diagnostic	1 (4)
Epidural blood patch	Therapeutic	1 (4)
Occipital nerve block	Therapeutic	1 (4)
Ophthalmic surgery (laser photocoagulation)	Therapeutic	1 (4)
Subcutaneous fat aspirate	Diagnostic	1 (4)

^a^ Procedures involving direct contact with central nervous system tissues were deemed high risk. Invasive procedures with disruption of mucosal or lymphoid tissues (eg, joint replacement and intra-abdominal laparotomy) were considered moderate risk. Procedures with minimal disruption of mucosal or lymphoid tissues (eg, endoscopy and colonoscopy) were considered low risk. Invasive procedures with disposable instruments only involving contact with blood were deemed no or negligible risk.^3,4^

**Table 2.** Demographic Characteristics, Symptoms and Signs at Presentation, and Results of Investigations for Patients With Probable or Definite CJD

Characteristic	Patients, No. (%)			*P* value
	Total (N = 121)	Underwent procedure (n = 26)	Did not undergo procedure (n = 95)	
Age at symptom onset, median (range), y	65.4 (21.9-81.5)	65.4 (21.9-78.9)	65.4 (32.3-81.5)	.58
Sex				
Female	63 (52)	16 (62)	47 (49)	.28
Male	58 (48)	10 (38)	48 (51)	
Race				
Black	2 (2)	1 (4)	1 (1)	.32
White, nonHispanic	111 (92)	23 (88)	88 (93)	.49
Other^a^	8 (7)	2 (8)	6 (6)	.80
Medical history				
Vascular risk factors	75 (62)	19 (73)	56 (59)	.19
Cataracts	7 (6)	2 (8)	5 (5)	.64
Peripheral neuropathy	1 (1)	0	1 (1)	.60
Arthritis	7 (6)	4 (15)	3 (3)	.02
Symptoms and signs at presentation				
Cognitive	105 (87)	20 (77)	83 (87)	.18
Behavioral	45 (37)	6 (23)	39 (41)	.09
Vision loss or change	26 (21)	5 (19)	20 (21)	.84
Sensorimotor	97 (80)	20 (77)	65 (67)	.40
Ataxia	82 (68)	17 (65)	75 (79)	.15
Constitutional	15 (12)	3 (12)	12 (13)	.88
Time from symptom onset to presentation, median (range), mo	1.9 (0-29.0)	2.6 (0.4-29.0)	1.7 (0-20.0)	.06
Results of investigations, No. of patients/total No. (%)				
Brain MRI consistent with CJD^b^	108/120 (90)	21/26 (81)	73/94 (78)	.73
Time from symptom onset to first MRI, median (range), mo	2.8 (0-36.7)	5.9 (0-36.7)	2.7 (0-30.3)	.05
Abnormal EEG	94/111 (85)	22/25 (88)	73/86 (85)	.70
Time from symptom onset to first EEG, median (range), mo	3.3 (0.3-34.6)	5.0 (0.8-34.6)	3.2 (0.3-23.1)	.07
CSF 14-3-3 positive	73/111 (66)	17/23 (74)	57/88 (65)	.41
Real-Time Quaking-Induced Conversion positive	87/95 (92)	20/21 (95)	66/73 (90)	.48
CSF total tau protein >1150 pg/mL	88/92 (96)	20/21 (95)	68/72 (94)	.89
Time from symptom onset to first CSF, median (range), mo	3.7 (0.4-37.4)	3.5 (0.4-37.4)	3.7 (0.4-23.4)	.34
Outcome data				
Deceased	109 (90)	21 (81)	88 (93)	.14
Symptomatic duration, median (range), mo^c^	5.6 (0.9-56.9)	9.3 (1.4-56.9)	5.5 (0.9-37.4)	.37

Abbreviations: CJD, Creutzfeldt-Jakob disease; CSF, cerebrospinal fluid; EEG, electroencephalogram; MRI, magnetic resonance imaging.

^a^ Other includes Asian, White Hispanic, or not reported.

^b^ Brain MRI criteria as defined within the Centers for Disease Control and Prevention Diagnostic Criteria (high signal in caudate or putamen or at least 2 cortical regions [temporal, parietal, occipital] either on diffusion-weighted imaging or fluid-attenuated inversion recovery).^5^

^c^ Data were available from 21 patients undergoing a procedure and for 88 patients without a history of a procedure.

## Discussion

Invasive procedures were frequently performed in patients with CJD included in this case-control study. Actual numbers of procedures may have been even greater, recognizing that procedures performed at outside hospitals may have been overlooked or excluded from records. Replication of study methods within additional hospitals—including community-based centers—is required to confirm these findings and establish the generalizability of results.

Features of sporadic CJD typically manifest in the sixth through eighth decades of life (median age, 68 years),^5^ a time when gait abnormalities, sensorimotor complaints and visual changes may be mistaken for common age-related surgically responsive conditions, leading to surgical procedures in this cohort and others.^6^ Thus, it is essential to accurately decipher the cause of symptoms and signs in clinical practice (eg, distinguishing between difficulty walking due to joint pain vs ataxia due to CJD). Preoperative risk assessment tools may identify patients at risk of CJD, for whom elective surgical procedures should be deferred and emergent procedures completed under precautions.^2^ However, prescreening cannot prevent invasive procedures in presymptomatic patients. National registries may address this problem. In the US, The National Prion Disease Pathology and Surveillance Center systematically collects data from patients with suspected CJD. Incorporating questions on recent invasive procedures would allow early notification of surgeons or facilities when a diagnosis of CJD is confirmed, permitting quarantine, decontamination, or decommissioning of affected instruments. This would also allow for prospective surveillance in larger numbers of patients, providing data needed to replicate our findings and quantify the scope of the potential problem posed by surgical procedures in patients with CJD.
